# Mass Cytometry Exploration of Immunomodulatory Responses of Human Immune Cells Exposed to Silver Nanoparticles

**DOI:** 10.3390/pharmaceutics14030630

**Published:** 2022-03-12

**Authors:** Jiwon Bae, My Ha, Haribalan Perumalsamy, Yangsoon Lee, Jaewoo Song, Tae-Hyun Yoon

**Affiliations:** 1 Department of Chemistry, College of Natural Sciences, Hanyang University, Seoul 04763, Korea; lovi109@hanyang.ac.kr (J.B.); my.ha@uantwerpen.be (M.H.); 2 Research Institute for Convergence of Basic Science, Hanyang University, Seoul 04763, Korea; harijai2004@gmail.com; 3 Department of Laboratory Medicine, College of Medicine, Hanyang University, Seoul 04763, Korea; yangsoon@hanyang.ac.kr; 4 Department of Laboratory Medicine, College of Medicine, Yonsei University, Seoul 03722, Korea; labdx@yuhs.ac; 5 Institute of Next Generation Material Design, Hanyang University, Seoul 04763, Korea; 6 Yoon Idea Lab Co., Ltd., Seoul 04763, Korea

**Keywords:** single-cell analysis, mass cytometry, silver nanoparticles, immune systems, immunomodulatory responses

## Abstract

Increasing production and application of silver nanoparticles (Ag NPs) have raised concerns on their possible adverse effects on human health. However, a comprehensive understanding of their effects on biological systems, especially immunomodulatory responses involving various immune cell types and biomolecules (e.g., cytokines and chemokines), is still incomplete. In this study, a single-cell-based, high-dimensional mass cytometry approach is used to investigate the immunomodulatory responses of Ag NPs using human peripheral blood mononuclear cells (hPBMCs) exposed to poly-vinyl-pyrrolidone (PVP)-coated Ag NPs of different core sizes (i.e., 10-, 20-, and 40-nm). Although there were no severe cytotoxic effects observed, ^PVP^Ag^10^ and ^PVP^Ag^20^ were excessively found in monocytes and dendritic cells, while ^PVP^Ag^40^ displayed more affinity with B cells and natural killer cells, thereby triggering the release of proinflammatory cytokines such as IL-2, IL-17A, IL-17F, MIP1β, TNFα, and IFNγ. Our findings indicate that under the exposure conditions tested in this study, Ag NPs only triggered the inflammatory responses in a size-dependent manner rather than induce cytotoxicity in hPBMCs. Our study provides an appropriate ex vivo model to better understand the human immune responses against Ag NP at a single-cell level, which can contribute to the development of targeted drug delivery, vaccine developments, and cancer radiotherapy treatments.

## 1. Introduction

Nanomaterials have been used widely in various industrial applications and consumer products, such as food additives, textiles, cosmetics, biosensors, and drug delivery carriers [1,2,3], which has raised concerns about the potential risks of nanoparticles to human health and the environment. Particularly, silver nanoparticles (Ag NPs) have been used widely in biomedical applications, such as drug carriers for target delivery, nanoprobes for disease diagnosis, bioimaging, and labeling agents for cell labeling and gene delivery [3]; or in consumer products, such as laundry additives, paints, and textiles due to their antiseptic properties [4]. However, extensive use of Ag NPs might pose a threat to human health and the environment [3,5,6]. For instance, Ag NPs can reach systemic circulation in the human body via different routes of administration, such as intravenous, nasal, oral, and cutaneous routes [7], and can induce aggregation of platelets and promote procoagulant activity in red blood cells [8,9,10]. They have also been reported to cause adverse biological effects such as oxidative stress, mitochondrial injury, DNA damage, cell-cycle arrest, and apoptosis induction [11,12,13,14]. Moreover, these NPs can be recognized by the human immune system as foreign pathogens and cause initiation of immune responses.

Among the various biomarkers of immunotoxicity, cytokine release is commonly used to study and predict immune responses. Cytokine release in the human body often causes mild symptoms such as fever, hypotension, nausea, headache, chills, vomiting, and muscle pain, but it can be life-threatening [15]. Hence, it is important to monitor NP-induced cytokine release in preclinical studies to understand, prevent, and control the immune responses to nanotherapeutics. Understanding the immune responses to metallic nanoparticle exposure is essential for understanding NP cytotoxicity and cell–NP interaction, and will help in the development of nanomaterials engineered for various biomedical purposes. Innate immunity is a primary concern due to its crucial role in upholding tissue and cellular homeostasis [16]. However, the interaction of NP with cells might alter or affect homeostasis of the immune system through recognition of foreign particles entering the body and stimulating immunological responses [17,18].

Under normal physiological conditions, the uptake of NPs by immune cells results in prolonged hyper- or hypoinflammatory immunological responses, which are recognized as a feature of nanomaterials used in biomedical applications [17,18,19]. A variety of quantitative and qualitative approaches have been employed to interrogate the various immunological expressions induced by exposure to NPs, such as inductively coupled plasma mass spectrometry (ICP-MS), flow cytometry, enzyme-linked immunosorbent assay (ELISA), and biological assays [20,21,22,23,24]. While numerous studies have been conducted on the interactions of immune systems with various NPs, very few studies have revealed the immunomodulatory responses of NPs in a holistic manner. This is primarily due to the highly heterogeneous nature of human immune systems interacting with the NPs, as well as the limited capability of the analytical tools to study these complex systems. To perform an in-depth study on these heterogeneous immune systems interacting with NPs, it is necessary to adopt a single-cell-based, high-dimensional approach, such as single-cell-based mass cytometry and RNA sequencing techniques [25,26,27]. Mass cytometry, or cytometry by time-of-flight (CyTOF), is a recently established technique that analyzes each single cell multiparametrically through multiple metal-tagged cellular markers with minimal overlap of signals [28]. Mass cytometry allows up to 50 lanthanide metal isotope labels, which overcomes the dimensional limitation of flow cytometry-based research about phenotyping and immune profiling of human peripheral blood mononuclear cells (hPBMCs).

In this study, we conducted mass cytometry experiments with hPBMCs exposed to PVP-coated silver nanoparticles (Ag NPs) of various sizes (10, 20, and 40 nm) to understand the immune responses of hPBMCs. For clearer and unbiased interpretations of high-dimensional mass cytometry data, we also adapted advanced visualization and clustering tools, such as Uniform Manifold Approximation and Projection (UMAP) and PhenoGraph clustering analysis. UMAP is a manifold learning technique for dimension reduction, while PhenoGraph is a robust computational method that can define phenotypes in high-dimensional single-cell data [29,30]. The cytokine-mediated intracellular immunological responses (either innate or adaptive) emphasized in a heterogeneous immune population were characterized by intracellular protein markers through mass cytometry analysis. Throughout this study, we have demonstrated that high-content data from mass cytometry can be used to improve our knowledge of how NPs interfere with the immune system. This information will provide useful guidance for the appropriate utilization of nanomaterials, especially Ag NPs, in commercial and biomedical applications.

## 2. Materials and Methods

### 2.1. Silver Nanoparticles

Polyvinylpyrrolidone (PVP)-coated Ag NPs with nominal diameters of 10, 20, and 40 nm (denoted as ^PVP^Ag^10^, ^PVP^Ag^20^, and ^PVP^Ag^40^, respectively) were purchased from NanoComposix (San Diego, CA, USA). The physicochemical characterizations (core size, hydrodynamic size, zeta potential, Ag dissolution ratio) of these Ag NPs were performed using TEM, DLS, and ICP-MS, and the results are detailed in Table S1 and Figure S1.

### 2.2. Isolation of PBMCs from Whole Blood

The human blood used in this study was obtained from Yonsei University Hospital (Seoul, Korea) with informed consent from the donors and approval from the Institutional Review Board (No. HYUH 2018-09-005-004). Human blood was drawn from healthy donors into Heparin-treated tubes (BD Vacutainer^®^, Franklin Lakes, NJ, USA), and PBMCs were isolated from whole blood via density-gradient centrifugation using Ficoll-Paque PLUS (GE Healthcare Bio-Sciences, Uppsala, Sweden). Briefly, blood was diluted 1:1 with phosphate-buffered saline (PBS) (Welgene, Gyeongsan-si, Korea), and the diluted blood was overlaid on the Ficoll reagent in centrifuge tubes. These tubes were centrifuged at 400× *g* at room temperature for 40 min in a centrifuge with a swing-bucket rotor (Labogene, Gimpo-si, Korea). The mononuclear layer was then collected and transferred to a new tube, washed in PBS, and pelletized by centrifugation at 200× *g* under room temperature for 10 min. The supernatant was discarded, and the cells were resuspended in RPMI-1640 complete media, (Lonza^TM^ BioWhittaker^TM^, Walkersville, MD, USA) supplemented with 10% fetal bovine serum (Gibco, Billings, MT, USA) and 1% penicillin/streptomycin (Gibco, MT, USA) for subsequent treatments. Triplicate measurements were performed with blood obtained from different donors for each replication to address interdonor variation.

### 2.3. Stimulation of PBMCs and Exposure to Ag NPs

PBMCs were stimulated with 5 ng/mL of phorbol 12-myristate 13-acetate (Sigma Aldrich, St. Louis, MA, USA) and 1 μg/mL of ionomycin (Sigma Aldrich, MA, USA) and treated with 2 μg/mL of Ag NPs for 3 h in the presence of protein transport inhibitors Brefeldin A (eBiosciences, Invitrogen, Waltham, MA, USA) and monensin (eBiosciences, Invitrogen, MA, USA). Cells were incubated at 37 °C with 5% CO_2_ in Petri dishes (SPL Life Sciences, Pocheon-si, Korea).

### 2.4. Surface and Intracellular Marker Staining

Cells were stained with surface and intracellular markers following the Maxpar Cytoplasmic/Secreted Antigen Staining with Fresh Fix Protocol (Fluidigm, USA). Briefly, cells were washed with PBS to remove excess Ag NPs and then stained with cisplatin for viability [31]. Subsequently, cells were stained with the surface markers listed in Table S2. After surface staining, cells were fixed in Maxpar Fix I Buffer (Fluidigm, South San Francisco, CA, USA) and permeabilized with Maxpar Perm-S Buffer (Fluidigm, CA, USA). Then, cells were stained with intracellular markers (Table S2). After intracellular staining, cells were fixed again with 1.6% formaldehyde and stained with Cell-ID Intercalator-Ir. Prior to data acquisition, cells were washed and suspended at 1 × 10^6^ cells/mL in Cell Acquisition Solution (Fluidigm, CA, USA). Calibration beads were added 1:10 by volume for normalization. Cells were then filtered into strainer-capped tubes and samples were analyzed on the Helios mass cytometry platform (Fluidigm, CA, USA).

### 2.5. Mass Cytometer Setup, Calibration, and Data Acquisition

A Helios mass cytometer (Fluidigm, CA, USA) was used for data acquisition. The instrument was tuned by optimizing the nebulizer, makeup gas, current, and detector voltage according to the manufacturer’s guidelines. For calibration of ^107^Ag counts, a blank solution (5% HNO_3_) and a AgNO_3_ solution (1 ng/mL in 5% HNO_3_) were measured in “solution mode”. The injection speed (or flow rate) was set to 5 × 10^−7^ L/s and the Push length was set to 13 s by default. The average dual counts of ^107^Ag in the collected data were used for calculating the cellular Ag NP association. To acquire data from the PBMC samples, the instrument was set to “event mode”.

### 2.6. Data Analysis

**Figure 1:**
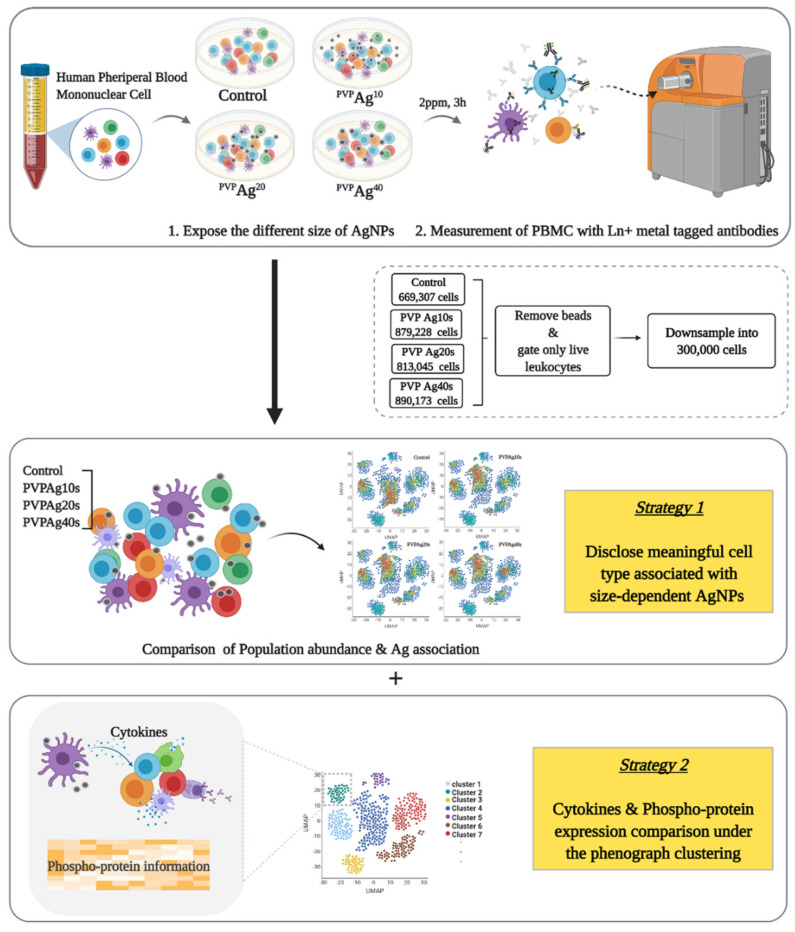
Schematic representation of analyzing pipeline of mass cytometry at single-cell resolution in human PBMC cells and dimensionality visualization performed by UMAP and PhenoGraph clustering to visualize data at single-cell resolution.

FlowJo (v10.7.2) (FlowJo, LLC, Ashland, Oregon, OR, USA) and Cytobank (v9.0) (Cytobank, Inc., Mountain View, CA, USA) were used for data gating and visualization. An inverse hyperbolic sine (arcsinh) transformation was applied to the raw data by applying the function: $x_{\mathrm{new}}=\arcsin\left(\sqrt[{2}]{x_{\mathrm{raw}}}\right)$. The cell populations were identified via manual gating (see Figure S2) based on the surface markers used in this study (Table S2). Quantification of cellular Ag NPs was accomplished following the method suggested by Ivask et al. [14]. The t-distributed stochastic neighbor embedding (t-SNE) and Uniform Manifold Approximation and Projection (UMAP) methods were used to visualize high-dimensional mass cytometry data at single-cell resolution. In addition to the manually gated phenotypes, PhenoGraph, an automated clustering method, was adapted to further identify cellular subsets and quantify their populations (Figure 1).

### 2.7. Statistical Analysis

The data are plotted using OriginPro software program (version 2016 b9.3.2.303 Academic, Origin Lab Corporation, Northampton, MA, USA). Statistical significance was assessed using the Mann–Whitney U test. 0.01 < *p* < 0.05, 0.001 < *p* < 0.01, *p* < 0.001 was considered significant for *, **, ***, respectively.

## 3. Results

### 3.1. Physicochemical Characteristics of Silver Nanoparticles

The physicochemical properties of the PVP-coated Ag NPs, such as core size, hydrodynamic size, zeta potential, and dissolution ratio, are summarized in Table S1 and Figure S1. To validate the sizes of the Ag NPs such as ^PVP^Ag^10^, ^PVP^Ag^20^, and ^PVP^Ag^40^ from the manufacturer (10.2 ± 1.6, 19.7 ± 3.2, and 39 ± 4 nm and hydrodynamic sizes 18.6, 37.3, and 58 nm), TEM and DLS were performed. TEM images shown in Figure S1A demonstrate that the Ag NPs are relatively uniform in their sizes and shapes. Hydrodynamic sizes of ^PVP^Ag^10^, ^PVP^Ag^20^, and ^PVP^Ag^40^ in DI water were measured to be 10 ± 2 nm, 21 ± 4 nm, and 39 ± 4 nm, respectively. Additionally, the hydrodynamic sizes of the Ag NPs in RPMI media were measured by DLS to monitor aggregation/agglomeration in cell-culture media. The hydrodynamic sizes measured in RPMI media showed a significant agglomeration, particularly for the Ag NPs with smaller core sizes. The hydrodynamic sizes of the ^PVP^Ag^10^ and ^PVP^Ag^20^ in RPMI media were measured to be 41 ± 13 nm and 75 ± 9 nm, respectively, about 4 times larger than their hydrodynamic sizes in DI water, whereas the hydrodynamic size of the ^PVP^Ag^40^ in RPMI media was found to be 77 ± 33 nm, less than 2 times its hydrodynamic size in DI water. As shown in Table S1, the absolute value of the zeta potentials (mV) of the Ag NPs are significantly reduced in the RPMI media compared to in DI water. Dissolution measurements were also performed using ICP-MS, since the dissolution of Ag NPs is an important factor for the evaluation of Ag^+^ ion influences and cellular doses of Ag NPs (Figure S1B). Our results indicate very small amounts of Ag were dissolved from all three NP sizes.

### 3.2. Cellular Association of Ag NPs on hPBMCs: Effects of Cell Type and NP Core Size

**Figure 2:**
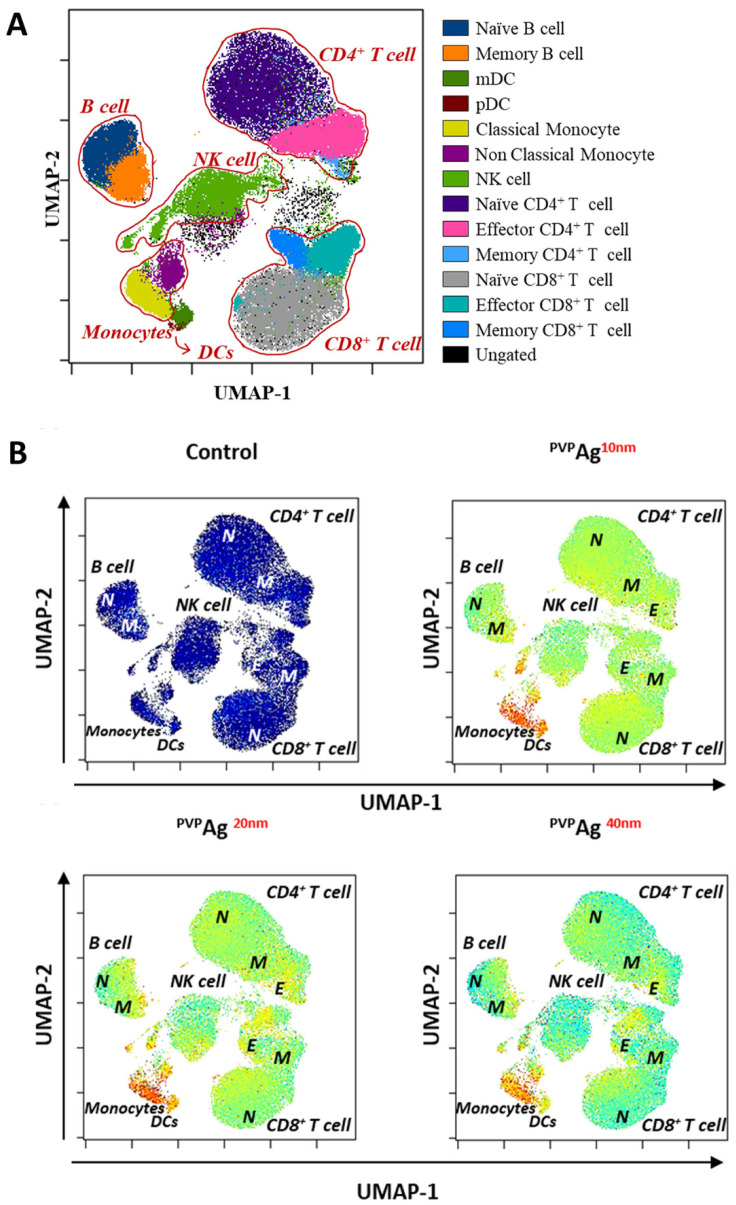
Manually gated cellular phenotypes. (**A**) Visualization of distinguished cellular types with manually gated islands; (**B**) Intensity of cellular interaction between ^PVP^Ag^10^, ^PVP^Ag^20^, and ^PVP^Ag^40^ Ag NPs and hPBMC overlaid on UMAP plots.

The cellular associations of Ag NPs were investigated using high-dimensional mass cytometry to study the effects of immune cell types and Ag NPs’ physicochemical properties. Based on our experiences from previous studies [32,33], exposure conditions (e.g., 2 µg/mL of exposure concentration and 3 h of exposure time) of the Ag NPs were chosen to avoid saturation of ^107^Ag signal intensity on the Helios platform and to maintain adequate cell viability while still ensuring noticeable changes to the cellular responses (Figure 1). Using the manual-gating strategy presented in Figure S2, 13 cell types were identified and assigned in the UMAP visualizations of the high-dimensional mass cytometry data shown in Figure 2A,B: classical and nonclassical monocytes; plasmacytoid and myeloid dendritic cells (i.e., pDCs and mDCs, respectively); natural killer (NK) cells; naïve and memory B cells; and naïve, effector, and memory CD4^+^ and CD8^+^ T cells. Similar to other nonlinear-dimensionality-reduction techniques (e.g., t-SNE and vi-SNE), UMAP is able to reduce the dimensionality of the data to visualize in a 2D plot. Additionally, UMAP is known to have the advantage of preserving the global data structure, while t-SNE has the drawback of losing intercluster (or global) information. Therefore, in the UMAP plot, cell types with similar characteristics are closely located, while distinct cell types are well separated from each other. For instance, B cells are in the upper left region, monocytes and DCs are in the lower left region, NK cells are in the central region, CD4^+^ T cells are in the upper right region, and CD8^+^ T cells are in the lower right region of the UMAP. The subsets of the CD4^+^ and CD8^+^ T cells, such as effector (denoted as E), memory (denoted as M), and naïve (denoted as N) cells, are located more closely than the other cell types, and the proximity of dendritic cell and monocytes, which are known to share many similarities, also demonstrate preservation of the intercluster information in the UMAP visualization.

**Figure 3:**
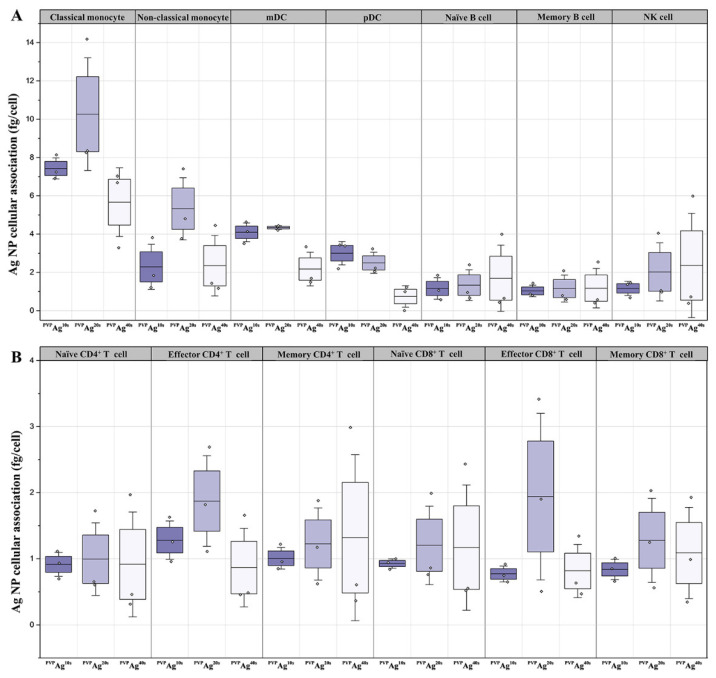
Cellular interaction of ^PVP^Ag^10^, ^PVP^Ag^20^, and ^PVP^Ag^40^ Ag NPS in hPBMC. (**A**) Comparison of the cellular association of non-T cells and (**B**) T-cell immune cell types are identified and manually gated population is shown in boxplots.

In Figure 2A, the amounts of cell-associated ^107^Ag were also overlaid on the UMAP plots. Significant cellular associations of Ag NPs were observed, especially for the monocytes and dendritic cells (DC), located in the bottom left corner of the UMAP plots. The red-colored islands of monocytes and DCs indicate higher levels of ^107^Ag in these cell types, whereas the other islands in the UMAP plots displayed blue and green colors, indicating low-to-medium cellular levels of ^107^Ag, respectively. However, although the cell-type dependance of the cellular association of Ag NPs can be easily found from visual inspection of the UMAP plots, the differences induced by Ag NPs with different core sizes were not clear upon visual inspection of the UMAP plots. Therefore, to support these qualitative data of human immune cells associated with Ag NPs based on UMAP visualization, we quantitatively compared the cellular uptake of Ag NPs with different core sizes in different immune cell types. The boxplots shown in Figure 3 confirm our observation from the UMAP plots. Phagocytic cells were typically associated with 2–14.2 femtograms of Ag per cell (fg/cell), which is much higher than the typical cellular association levels of Ag with other cell types (0–3 fg/cell) (Figure 3). Furthermore, ^PVP^Ag^20^ displayed around 1.5–2.5-fold higher association with classical and nonclassical monocytes, compared with ^PVP^Ag^10^ and ^PVP^Ag^40^; and ^PVP^Ag^10^ and ^PVP^Ag^20^ were more highly accumulated in both types of myeloid and plasmatoid dendritic cells (mDC, pDC) than the largest nanoparticle, ^PVP^Ag^40^. On the other hand, naïve B cells and NK cells showed an inclined gradient in cellular uptake with different sizes of Ag NPs (Figure 3). It could be assumed that the extracellular or membrane-bounded Ag NPs were engulfed by NK cells followed by the cellular stress response considered to be cytotoxic, or by B cells with some phagocytic properties. However, for the other cell types (e.g., CD4^+^ and CD8^+^ T cells), there was hardly any significant size-dependent trend in their AgNP uptake.

### 3.3. Variations of Immune Cell Populations: Effect of the Core Sizes of Ag NPs

**Figure 4:**
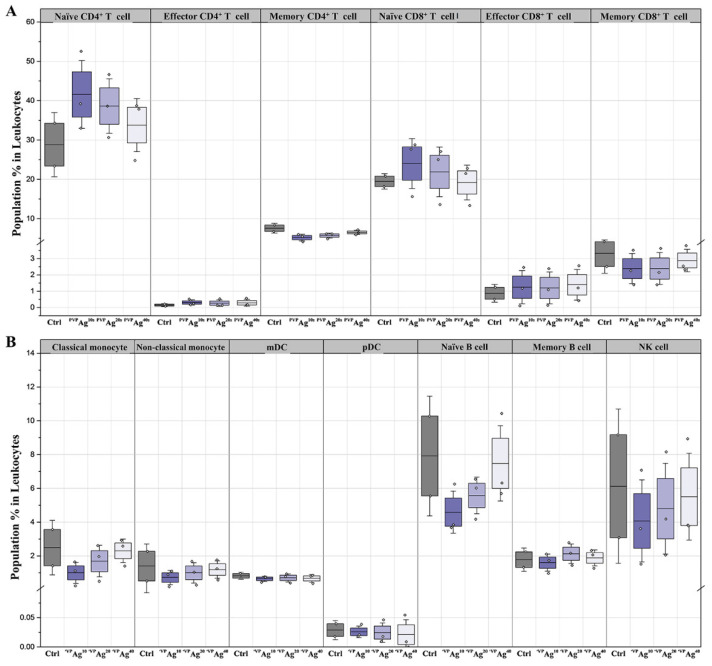
Comparison of the population of (**A**) T cells and (**B**) non-T cells in samples exposed to ^PVP^Ag^10^, ^PVP^Ag^20^, and ^PVP^Ag^40^ Ag NPs.

The variation between different cell populations in their immune response against the exposure to ^PVP^Ag^10^, ^PVP^Ag^20^, and ^PVP^Ag^40^ was also investigated using mass cytometry. As shown in Figure 4, the smaller Ag NPs (i.e., ^PVP^Ag^10^, ^PVP^Ag^20^) caused a significant reduction of innate immune cell populations (e.g., classical/nonclassical monocytes, naïve B cells and NK cells), whereas the larger-sized Ag NPs ^PVP^Ag^40^ showed significant increment of adaptive immune cell types, including naïve CD4^+^ T and CD8^+^ T cells, compared with the untreated control.

In the case of monocytes, the classical monocytes contributed 1.0, 1.6, and 2.3%, whereas nonclassical monocytes contributed 0.7, 1.0, and 1.2% of the total leukocyte populations for the samples exposed to ^PVP^Ag^10^, ^PVP^Ag^20^, and ^PVP^Ag^40^, respectively. Interestingly, the samples exposed to the smaller Ag NPs displayed reduced cellular abundances compared to the untreated control sample, whereas the sample exposed to the larger Ag NPs (i.e., ^PVP^Ag^40^) displayed similar levels of cellular abundance as the control sample. As described in the previous section (Figure 3), since monocytes are phagocytic cells, they have a greater tendency to ingest foreign particles than nonphagocytic cells [34,35], and smaller Ag NPs were easily engulfed by membrane transfusion. Higher association with the smaller Ag NPs may cause apoptosis, resulting in decreases to monocyte populations (Figure 4). Similarly, for the samples exposed to the ^PVP^Ag^10^, ^PVP^Ag^20^, and ^PVP^Ag^40^, the percentage of the populations consisting of naïve B cells were found to be 4.5, 5.6, and 7.5%, which are 1.7-, 1.4-, and 1.1-fold lower than those of the control sample, and the percentage of the populations consisting of NK cells were found to be 4.1, 4.7, and 5.5%, which are 1.5-, 1.3-, and 1.1-fold lower than those of the control sample (Figure 4). As discussed previously, the cellular uptake of large-sized Ag NPs (i.e., ^PVP^Ag^40^) found on naïve B cells and NK cells initiated innate cellular responses with a particle size-dependent manner.

The populations of naïve CD4^+^ T cells were 1.4-, 1.3-, and 1.2-fold higher in the samples exposed to ^PVP^Ag^10^, ^PVP^Ag^20^, and ^PVP^Ag^40^; and those of naïve CD8^+^ T cells were 1.2- and 1.1-fold higher only in ^PVP^Ag^10^, ^PVP^Ag^20^; and no significant fold changes were found in ^PVP^Ag^40^, respectively, than those of the control sample. Similarly, we also observed that the APCs, such as mDC and pDC, are highly associated with the smaller Ag NPs (i.e., ^PVP^Ag^10^ and ^PVP^Ag^20^) (Figure 3) and that a percentage of the population of naïve T cells (i.e., naïve CD4^+^ and naïve CD8^+^ T cells) showed significant increases when they were exposed to the smaller Ag NPs (Figure 4).

### 3.4. Variations of PhenoGraph Subclusters of Immune Cell Populations: Effect of the Core Sizes of Ag NPs

**Figure 5:**
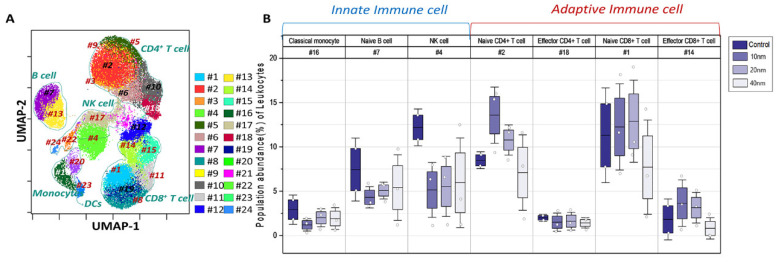
Population abundance of PhenoGraph clusters of immune cells. (**A**) PhenoGraph cluster-overlaid UMAP (**B**) Significant immune-subset cluster population shown in boxplots.

In addition to the manual gating, an automated clustering approach, such as the PhenoGraph clustering algorithm, was applied to discover cellular subsets and monitor their immunomodulatory responses to the Ag NP exposures. A total of 24 cellular subsets were identified by the PhenoGraph clustering algorithm (Figure 5A and Figure S3 and Table 1), and most of these clusters were matched to manually gated cell types (Figure 2A). The PhenoGraph-based clusters were identified as subsets of both innate and adaptive immune cell populations (Figure 5A,B)—in particular, four subset populations from the NK cell type, naïve CD4^+^ T cells, two subset clusters of memory CD4^+^ T cells, and five clusters as subsets of naïve CD8^+^ T cells. The other cell types, such as the effector CD4^+^ T cell, effector/memory CD8^+^ T cell, naïve/memory B cell, and classical/nonclassical monocytes, were clustered as one type (Figure S5). Among the cellular subsets, the significant cell types were identified as classical monocytes (#16), naïve B cells (#7), NK cells (#4), naïve CD4^+^ T cells (#2), effector CD4^+^ T cell (#18), naïve CD8^+^ T cell (#1) and effector CD8^+^ T cells (#14), respectively (Figure 5B). Based on this automated clustering of cellular subsets, we further studied immunomodulatory responses of cellular subsets exposed to Ag NPs with different sizes.

### 3.5. Variations of in Intracellular Cytokine Expression: Effect of the Core Size of Ag NPs

**Figure 6:**
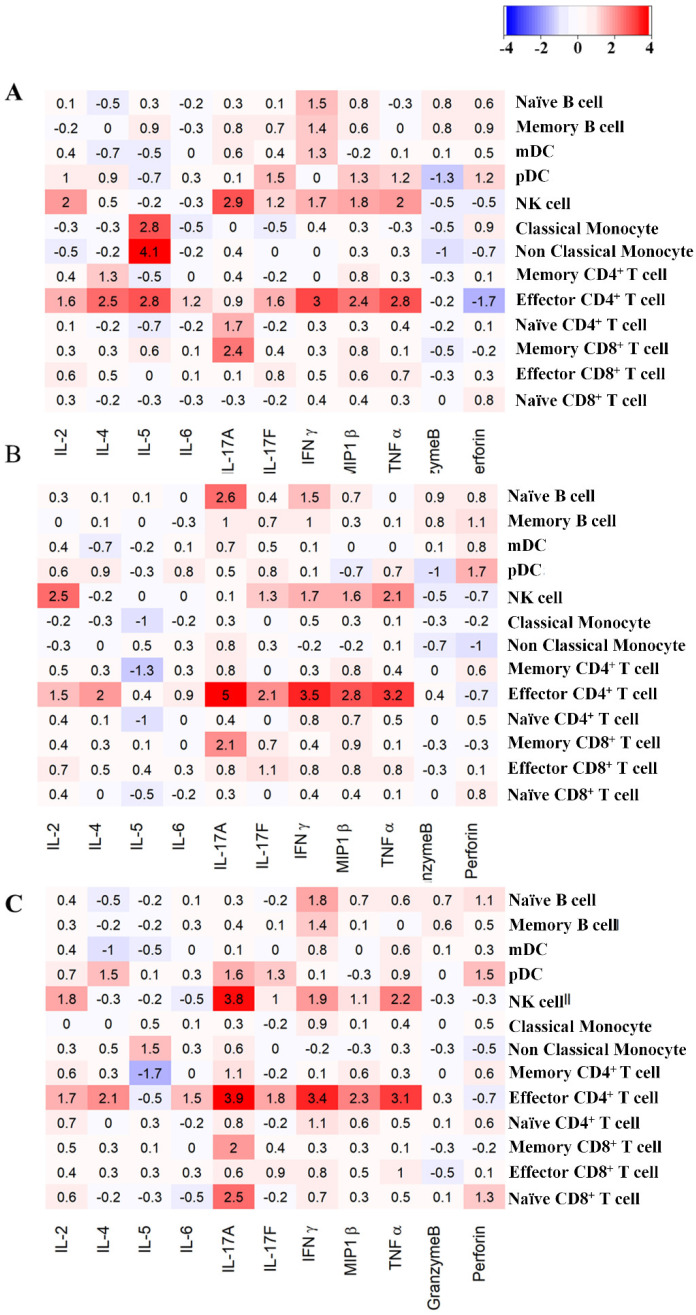
The intracellular cytokine-mediated expression. The heat map represents log_2_-fold expression of intracellular cytokines of different immune cell types when exposed to (**A**) ^PVP^Ag^10^ (**B**) ^PVP^Ag^20^, and (**C**) ^PVP^Ag^40^.

As shown in Figure 6, variations in intracellular cytokine expression for the manually gated hPBMC cell types were analyzed and visualized as a heatmap. The number inside each box is the log_2_ expression ratio of 11 cytokines in 13 immune cell types exposed to AgNPs of different sizes compared with the untreated control sample. The red and blue colors in the heatmap represent the numerical data of up- and downregulations of cytokines, and the color scale used blue to red, representing lower- and higher-value data points (−4 for blue and 4 for red). In NK cells, proinflammatory cytokines, such as IL-2, IL-17A, IL-17F, MIP1β, TNFα, and IFNγ, were significantly upregulated upon exposure to all ^PVP^Ag^10^, ^PVP^Ag^20^, and ^PVP^Ag^40^ NPs.

Interestingly, in the samples exposed to Ag NPs of different sizes, the TNFα and IFNγ cytokines displayed slight increments in fold changes (^PVP^Ag^10^: 2.0 and 1.7, ^PVP^Ag^20^: 2.1 and 1.7 and ^PVP^Ag^40^: 2.2 and 1.9) as the previously described cellular abundances of NK cells (Figure 4). In contrast, there are slight changes in MIP1β cytokine, which displayed significant fold changes in the samples exposed to smaller Ag NPs (^PVP^Ag^10^: 1.8, ^PVP^Ag^20^: 1.6) than the largest Ag NP (^PVP^Ag^40^: 1.1). The log_2_-fold expression pattern of IL-17A, IL-17F, MIP1β, TNFα, and IFNγ in effector CD4^+^ T cells increased slightly more in samples exposed to ^PVP^Ag^10^ (0.9, 1.6, 2.4, 2.8 and 3) and ^PVP^Ag^20^ (5, 2.1, 2.8, 3.2 and 3.5) than in those treated with ^PVP^Ag^40^ (3.8, 1, 1.1, 2.2 and 1.9). This finding confirms the cellular association of Ag NPs with effector CD4^+^ T cells, as previously described in Figure 3. However, there are no significant changes observed in the cellular abundance of effector CD4^+^ T cells in all exposure conditions (Figure 4). The cell viability was observed by cisplatin uptake following exposure of Ag NPs, denoting that most of the cells remain substantially viable (Figure S4).

## 4. Discussion

Physicochemical properties of nanoparticles are known to affect their toxicity and other interactions with cells. Previous literature reporting that smaller NPs can become more highly agglomerated than the larger NPs due to their high surface reactivity, which tends to form larger clusters in order to reduce their surface energy and increase stability [36,37,38,39,40]. In addition, under the same mass-dose condition, there is a higher number of particles per volume of smaller NPs than of larger NPs, so the higher collision probability of the smaller NPs may facilitate NP aggregation/agglomeration [36,37,41,42]. Our data on the zeta potentials indicate that the high ionic strength and proteins in RPMI media play an important role in determining the Ag NPs physicochemical properties in RPMI media and that these properties are different for the Ag NPs with smaller (i.e., ^PVP^Ag^10^ and ^PVP^Ag^20^) and larger (i.e., ^PVP^Ag^40^) core sizes (Table S1). Additionally, the ICP-MS data indicate very small amounts of Ag were dissolved from all three NP sizes. This dissolution analysis could be attributed to the protein corona of Ag NPs by FBS protein of RPMI media [43]. Reduction in the dissolution of Ag NPs due to the presence of serum proteins has also been reported [37,43,44]. These studies suggest that proteins adsorbed on the surface of Ag NPs may block oxidation, thus slowing the release of Ag^+^ ions. Our results also confirm the size-dependent dissolution of NPs reported by previous studies, in which dissolution ratio decreases as the NP’s size increases, and if the NP is large enough, ion dissolution rarely occurs [37,45]. Though the dissolution amounts are small, our results show that ^PVP^Ag^10^ and ^PVP^Ag^20^ have 10 times higher dissolution ratios than ^PVP^Ag^40^. Dissolution of the Ag NPs is known to be strongly affected by the size of the NP as well as the composition of the surrounding medium [38,46,47,48], and our studies are consistent with these results. Overall, the low dissolution ratio of the Ag NPs used in this study showed that cellular Ag association, either estimated or measured, can be assumed to be due to the cellular internalization of Ag NPs, rather than of dissolved Ag^+^ ions. The physicochemical properties of Ag NPs may play crucial roles throughout the processes of cellular uptake, intercellular trafficking, and cytotoxicity.

Numerous studies have reported the size dependence of the cellular association of NPs. Among their various physicochemical properties, the core size of a nanoparticle is known to play an important role in determining the process of its cellular uptake [49]. For instance, the internalization of smaller NPs consumes less energy and therefore happens more easily than it does for larger NPs. NPs with sizes ranging from 10 to 30 nm may actively cross the cell membrane, whereas internalization of NPs of larger sizes requires a passive mechanism, such as endocytosis [50]. Previous studies [51,52,53,54] have also suggested that smaller nanoparticles, such as ^PVP^Ag^10^ and ^PVP^Ag^20^ used in this study, undergo membrane transfusion or micropinocytosis cellular uptake through nonspecific internalization through innate immune cells (e.g., monocytes and dendritic cells). In contrast, larger nanoparticles, such as ^PVP^Ag^40^ used in this study, are known to undergo endocytosis, either via the clathrin- or caveolae-dependent mechanism or the micropinocytosis process of cellular internalization through innate immune cells, including naïve B cells, NK cells, monocytes, and DCs [54,55,56]. Our observations confirm that cellular association of Ag NPs in hPBMCs are strongly dependent on both physicochemical properties (i.e., the Ag NPs core size) and cell types (e.g., phagocytic or nonphagocytic cells).

**Figure 7:**
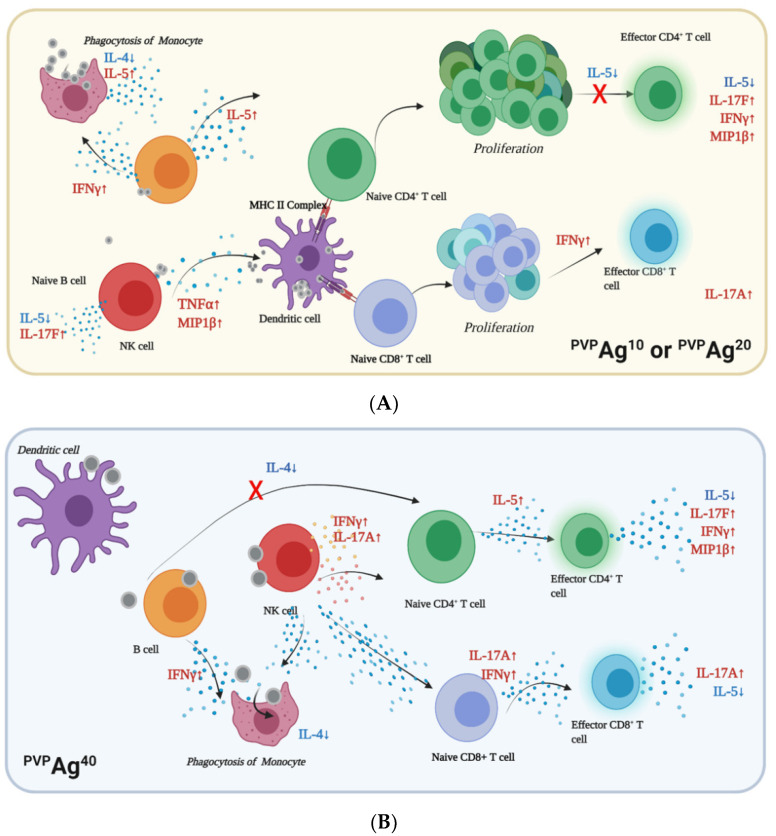
Illustration of immunomodulatory responses of hPBMCs exposed to (**A**) the smaller ^PVP^Ag^10^ and ^PVP^Ag^20^ and (**B**) the larger ^PVP^Ag^40^.

Significant cellular associations of Ag NPs were observed in monocytes and dendritic cells (DC), which is in good agreement with the previous studies [32,33] and current understanding of the cellular association of NPs; since monocytes are prominent antigen-presenting cells (APCs), they have a natural tendency to ingest foreign particles via phagocytosis [57,58]. Previous studies [34,55] have suggested that antigen-presenting cells recognize nanoparticles as foreign antigens, engulf and process them, then present them to effector cells via the major histocompatibility complex, thereby priming the antigen-specific cellular immune response. The smaller Ag NPs, ^PVP^Ag^10^ and ^PVP^Ag^20^, were preferentially associated with APCs, such as monocytes and DCs, to initiate immune responses. Previous studies suggest that naïve CD4^+^/CD8^+^ T cells proliferate and differentiate rapidly when they are primed by APCs [59,60]. Their proliferation and differentiation result in effector and memory T cells, as well as their antigen-specific T-cell receptors that can recognize and exert immune response against Ag NPs [35,61]. Furthermore, our data show that the PVP-coated Ag NPs, although taken up in considerable quantity by naïve, effector, or memory CD4^+^ and CD8^+^ T-cells, do not have significantly adverse effects on the number or viability of T-cells. These PVP-coated Ag NPs are therefore minimally hazardous to the adaptive immune response and can potentially be used in T-cell-targeted immunotherapy (Figure 7A,B).

In NK cells, proinflammatory cytokines, such as IL-2, IL-17A, IL-17F, MIP1β, TNFα, and IFNγ, were significantly upregulated upon exposure to Ag NPs of different sizes (10–40 nm). NK cells are members of the innate immunity, which provides the initial immune responses. This explains why the cytokine secretion of NK cells was much more significant than that of their adaptive immune counterpart, effector CD8^+^ T cells, which would need antigen-specific stimulation to elicit immune responses. Among the proinflammatory cytokines, the TNFα and IFNγ secreted by NK cells were previously reported to induce the elimination of infected cells or activate macrophages (or monocytes in the peripheral blood) to kill the phagocytosed microbes [62,63,64]. The NK cells secreted TNFα and IFNγ to promote inflammation and activate monocytes to eliminate the phagocytosed Ag NPs. The MIP1β is also known as a chemokine that promotes migration when cells are exposed to a stimulus. Previously, it was reported as the chemokine responsible for T-cell recruitment, which initiates antigen-specific responses in adaptive immunity [65,66]. On the other hand, Granzyme B and perforin were not significantly released, particularly in NK cells. The secretion of these cytokines helps NK cells initiate the apoptosis process in infected cells in the case of viral or bacterial infections [63]. In this study, the absence of granzyme B and perforin—as well as the presence of IFNγ—in NK cells indicate that the immune response against Ag NPs did not depend on the ‘killing’ of cells having high Ag NP association, but rather on the activation of monocytes to decompose the Ag NPs in phagolysosomes. Effector CD4^+^ T cells are members of the adaptive immunity, which provides antigen-specific immune responses that happen later than innate immune responses. Therefore, the secretion of IL-17A, IL-17F, MIP1β, TNFα, and IFNγ in effector CD4^+^ T cells following their exposure to Ag NPs promotes immunomodulatory inflammation [67]. Recently, the immunomodulatory effect of NPs has gained the interest of scientists in cancer research, and metallic NPs have been exploited as radio-sensitizing agents in radiotherapy [68]. We proved in the current study that Ag NPs have immunomodulatory effects in human peripheral blood immune cells. It would be interesting to also examine their ability to inhibit thioredoxin reductase enzymes, which play a key role in radiotherapy [69,70]. The methodology presented in our study opens new potential for examining the enzymatic inhibition ability of metallic NPs at a single-cell level and further applications of NPs in cancer treatments.

## 5. Conclusions

Our study provides an appropriate ex vivo model for predicting heterogeneous immune responses in human PBMC at the single-cell level though mass cytometry analysis. Herein, we uncovered in-depth knowledge of the silver nanoparticle’s interaction with immune cells that can be exploited in the design of nanomaterials with engineered immunomodulatory properties for future clinical application. The methodology presented here can be further applied on other metallic NPs to investigate their immunomodulatory effects to be utilized as drug carriers or radio-sensitizing agents in cancer treatments.

## Figures and Tables

**Table 1:** List of cellular phenotypic surface markers.

Cell Type	Phenograph Cluster	Marker Expression
Naïve CD4^+^ T cell	#2	CD3^+^CD4^+^CD27^+^CD38^+^CD45RA^+^
	#3	CD3^+^CD4^+^CD27^+^CD38^+^CD45RA^+^
	#5	CD3^+^CD4^+^CD27^+^CD38^+^CD45RA^+^
	#9	CD3^+^CD4^+^CD27^+^CD38^+^CD45RA^+^
Effector CD4^+^ T cell	#18	CD3^+^CD4^+^CD27^-^CD38^-^CD45RA^-^
Memory CD4^+^ T cell	#6	CD3^+^CD4^+^CD27^+^CD38^+^CD45RA^-^
	#10	CD3^+^CD4^+^CD27^+^CD38^-^CD45RA^-^HLA^-^DR^mid^
Naïve CD8^+^ T cell	#1	CD3^+^CD8^+^CD27^+^CD38^+^CD45RA^+^
	#8	CD3^+^CD8^+^CD27^+^CD38^+^CD45RA^+^
	#11	CD3^+^CD8^+^CD27^+^CD38^-^CD45RA^+^
	#19	CD3^+^CD8^+^CD27^+^CD38^mid^CD45RA^+^
	#21	CD3^+^CD8^+^CD27^+^CD38^mid^CD45RA^+^
Effector CD8^+^ T cell	#14	CD3^+^CD8^+^CD27^+^CD38^-^CD45RA^+^
Memory CD8^+^ T cell	#15	CD3^+^CD8^+^CD27^+^CD38^mid^CD45RA^-^HLA^-^DR^mid^
Naïve B cell	#7	CD19^+^CD20^+^CD27^-^CD38^+^CD45RA^+^HLA^-^DR^+^
Memory B cell	#13	CD19^+^CD20^+^CD27^+^CD38^+^CD45RA^+^HLA^-^DR^+^CD16^mid^CD11c^+^
NK cell	#4	CD19^-^CD8a^+^CD27^-^CD38^+^CD45RA^+^CD11c^+^
	#17	CD19^-^CD8a^+^CD27^mid^CD38^+^CD45RA^+^
	#22	CD19^-^CD8a^-^CD27^-^CD38^+^CD45RA^-^HLA^-^DR^+^CD11c^+^CD123^+^
	#24	CD19^-^CD8a^-^CD27^-^CD38^+^CD45RA^+^HLA^-^DR^+^CD123^+^
Classical monocyte	#16	CD14^+^CD11c^+^CD123^mid^HLA^-^DR^+^CD38^+^
Nonclassical monocyte	#20	CD14^-^CD11c^+^CD123^mid^HLA^-^DR^+^CD38^-^CD66a^+^
Dendritic cell	#23	CD11c^+^CD123^+^HLA^-^DR^+^CD38^+^
Unassigned	#12	CD3^+^CD4^+^CD27^+^CD38^-^CD45RA^+^

## Data Availability

The data presented in this study are available on request from the corresponding author.

## Supplementary Materials

The following supporting information can be downloaded at: https://www.mdpi.com/article/10.3390/pharmaceutics14030630/s1, Figure S1: Physicochemical characteristics of ^PVP^Ag^10^, ^PVP^Ag^20^, and ^PVP^Ag^40^; Figure S2: Manual gating strategies for identifying the major immune cell types; Figure S3: Intensity of phenotypic surface markers expression; Figure S4: The cell viability was observed by cisplatin uptake following exposure of Ag NPs; Figure S5: Immune cells subsets population of Population abundance of PhenoGraph clusters; Table S1: Hydrodynamic diameters and zeta potentials of the Ag NPs in deionized water and RPMI media with 10% FBS; Table S2: List of metal-tagged antibodies of surface markers and intracellular cytokine markers.

## References

[1] Flores A.M., Ye J., Jarr K.-U., Hosseini-Nassab N., Smith B.R., Leeper N.J. (2019). Nanoparticle Therapy for Vascular Diseases. Arterioscler. Thromb. Vasc. Biol..

[2] Kreuter J., Gelperina S. (2008). Use of Nanoparticles for Cerebral Cancer. Tumori J..

[3] Johnston B.D., Scown T.M., Moger J., Cumberland S.A., Baalousha M., Linge K., van Aerle R., Jarvis K., Lead J.R., Tyler C.R. (2010). Bioavailability of Nanoscale Metal Oxides TiO2, CeO2, and ZnO to Fish. Environ. Sci. Technol..

[4] Vance M.E., Kuiken T., Vejerano E.P., McGinnis S.P., Hochella M.F., Rejeski D., Hull M.S. (2015). Nanotechnology in the real world: Redeveloping the nanomaterial consumer products inventory. Beilstein J. Nanotechnol..

[5] Baker C., Pradhan A., Pakstis L., Pochan Darrin J., Shah S.I. (2005). Synthesis and Antibacterial Properties of Silver Nanoparticles. J. Nanosci. Nanotechnol..

[6] Samberg M.E., Orndorff P.E., Monteiro-Riviere N.A. (2011). Antibacterial efficacy of silver nanoparticles of different sizes, surface conditions and synthesis methods. Nanotoxicology.

[7] Elsabahy M., Wooley K.L. (2013). Cytokines as biomarkers of nanoparticle immunotoxicity. Chem. Soc. Rev..

[8] Greulich C., Diendorf J., Simon T., Eggeler G., Epple M., Köller M. (2011). Uptake and intracellular distribution of silver nanoparticles in human mesenchymal stem cells. Acta Biomater..

[9] Jun L., Qiuzhen W., Qingguo M. (2011). The effects of project uncertainty and risk management on IS development project performance: A vendor perspective. Int. J. Proj. Manag..

[10] Bian Y., Kim K., Ngo T., Kim I., Bae O.N., Lim K.M., Chung J.H. (2019). Silver nanoparticles promote procoagulant activity of red blood cells: A potential risk of thrombosis in susceptible population. Part. Fibre Toxicol..

[11] AshaRani P.V., Low Kah Mun G., Hande M.P., Valiyaveettil S. (2009). Cytotoxicity and Genotoxicity of Silver Nanoparticles in Human Cells. ACS Nano.

[12] Ghosh M., Manivannan J., Sinha S., Chakraborty A., Mallick S.K., Bandyopadhyay M., Mukherjee A. (2012). In vitro and in vivo genotoxicity of silver nanoparticles. Mutat. Res..

[13] Martinez-Gutierrez F., Thi E.P., Silverman J.M., de Oliveira C.C., Svensson S.L., Vanden Hoek A., Sánchez E.M., Reiner N.E., Gaynor E.C., Pryzdial E.L.G. (2012). Antibacterial activity, inflammatory response, coagulation and cytotoxicity effects of silver nanoparticles. Nanomedicine.

[14] Ivask A., Kurvet I., Kasemets K., Blinova I., Aruoja V., Suppi S., Vija H., Käkinen A., Titma T., Heinlaan M. (2014). Size-dependent toxicity of silver nanoparticles to bacteria, yeast, algae, crustaceans and mammalian cells in vitro. PLoS ONE.

[15] Shimabukuro-Vornhagen A., Gödel P., Subklewe M., Stemmler H.J., Schlößer H.A., Schlaak M., Kochanek M., Böll B., von Bergwelt-Baildon M.S. (2018). Cytokine release syndrome. J. Immunother. Cancer.

[16] Paradiso A., Domingo G.C., Blanco E., Buscaglia A., Fortunato S., Marsoni M., Scarcia P., Caretto S., Vannini C., de Pinto M.C. (2020). Cyclic AMP mediates heat stress response by the control of redox homeostasis and ubiquitin-proteasome system. Plant Cell Environ..

[17] Alijagic A., Gaglio D., Napodano E., Russo R., Costa C., Benada O., Kofroňová O., Pinsino A. (2020). Titanium dioxide nanoparticles temporarily influence the sea urchin immunological state suppressing inflammatory-relate gene transcription and boosting antioxidant metabolic activity. J. Hazard. Mater..

[18] Boraschi D., Italiani P., Palomba R., Decuzzi P., Duschl A., Fadeel B., Moghimi S.M. (2017). Nanoparticles and innate immunity: New perspectives on host defence. Semin. Immunol..

[19] Auguste M., Lasa A., Pallavicini A., Gualdi S., Vezzulli L., Canesi L. (2019). Exposure to TiO2 nanoparticles induces shifts in the microbiota composition of Mytilus galloprovincialis hemolymph. Sci. Total Environ..

[20] Hawkins S.J., Crompton L.A., Sood A., Saunders M., Boyle N.T., Buckley A., Minogue A.M., McComish S.F., Jiménez-Moreno N., Cordero-Llana O. (2018). Nanoparticle-induced neuronal toxicity across placental barriers is mediated by autophagy and dependent on astrocytes. Nat. Nanotechnol..

[21] Savage D.T., Hilt J.Z., Dziubla T.D. (2019). In Vitro Methods for Assessing Nanoparticle Toxicity. Methods Mol. Biol..

[22] Fraser J.A., Kemp S., Young L., Ross M., Prach M., Hutchison G.R., Malone E. (2018). Silver nanoparticles promote the emergence of heterogeneic human neutrophil sub-populations. Sci. Rep..

[23] Panzarini E., Mariano S., Vergallo C., Fimia G.M., Dini L., Mura F., Rossi M., Serra A., Casciaro S. Glucose capped silver nanoparticles enter HeLa cells and induce S and G2/M arrest. Proceedings of the 2015 1st Workshop on Nanotechnology in Instrumentation and Measurement (NANOFIM).

[24] Li L., Fernández-Cruz M.L., Connolly M., Conde E., Fernández M., Schuster M., Navas J.M. (2015). The potentiation effect makes the difference: Non-toxic concentrations of ZnO nanoparticles enhance Cu nanoparticle toxicity in vitro. Sci. Total Environ..

[25] Henriksen-Lacey M., Carregal-Romero S., Liz-Marzán L.M. (2017). Current Challenges toward In Vitro Cellular Validation of Inorganic Nanoparticles. Bioconjug. Chem..

[26] Kroll A., Pillukat M.H., Hahn D., Schnekenburger J. (2009). Current in vitro methods in nanoparticle risk assessment: Limitations and challenges. Eur. J. Pharm. Biopharm..

[27] Monteiro-Riviere N.A., Inman A.O., Zhang L.W. (2009). Limitations and relative utility of screening assays to assess engineered nanoparticle toxicity in a human cell line. Toxicol. Appl. Pharmacol..

[28] Bandura D.R., Baranov V.I., Ornatsky O.I., Antonov A., Kinach R., Lou X., Pavlov S., Vorobiev S., Dick J.E., Tanner S.D. (2009). Mass cytometry: Technique for real time single cell multitarget immunoassay based on inductively coupled plasma time-of-flight mass spectrometry. Anal. Chem..

[29] McInnes L., Healy J., Melville J. (2020). UMAP: Uniform Manifold Approximation and Projection for Dimension Reduction. arXiv.

[30] Levine J.H., Simonds E.F., Bendall S.C., Davis K.L., Amir E.D., Tadmor M.D., Litvin O., Fienberg H.G., Jager A., Zunder E.R. (2015). Data-Driven Phenotypic Dissection of AML Reveals Progenitor-like Cells that Correlate with Prognosis. Cell.

[31] Fienberg H.G., Simonds E.F., Fantl W.J., Nolan G.P., Bodenmiller B. (2012). A platinum-based covalent viability reagent for single-cell mass cytometry. Cytom. A.

[32] Ha M.K., Kwon S.J., Choi J.S., Nguyen N.T., Song J., Lee Y., Kim Y.E., Shin I., Nam J.W., Yoon T.H. (2020). Mass Cytometry and Single-Cell RNA-seq Profiling of the Heterogeneity in Human Peripheral Blood Mononuclear Cells Interacting with Silver Nanoparticles. Small.

[33] Ha M.K., Choi J.-S., Kwon S.J., Song J., Lee Y., Kim Y.-E., Yoon T.H. (2020). Mass cytometric study on the heterogeneity in cellular association and cytotoxicity of silver nanoparticles in primary human immune cells. Environ. Sci. Nano.

[34] Gerner M.Y., Torabi-Parizi P., Germain R.N. (2015). Strategically Localized Dendritic Cells Promote Rapid T Cell Responses to Lymph-Borne Particulate Antigens. Immunity.

[35] Curtsinger J.M., Schmidt C.S., Mondino A., Lins D.C., Kedl R.M., Jenkins M.K., Mescher M.F. (1999). Inflammatory Cytokines Provide a Third Signal for Activation of Naive CD4+ and CD8+ T Cells. J. Immunol..

[36] Ramachandran G., Kumar A.K.H., Kannan T., Bhavani P.K., Kumar S.R., Gangadevi N.P., Banurekha V., Sudha V., Venkatesh S., Ravichandran N. (2016). Low Serum Concentrations of Rifampicin and Pyrazinamide Associated with Poor Treatment Outcomes in Children with Tuberculosis Related to HIV Status. Pediatr. Infect. Dis. J..

[37] Ha M.K., Shim Y.J., Yoon T.H. (2018). Effects of agglomeration on in vitro dosimetry and cellular association of silver nanoparticles. Environ. Sci. Nano.

[38] Minghetti M., Schirmer K. (2016). Effect of media composition on bioavailability and toxicity of silver and silver nanoparticles in fish intestinal cells (RTgutGC). Nanotoxicology.

[39] Walczyk D., Bombelli F.B., Monopoli M.P., Lynch I., Dawson K.A. (2010). What the Cell “Sees” in Bionanoscience. J. Am. Chem. Soc..

[40] Monopoli M.P., Walczyk D., Campbell A., Elia G., Lynch I., Baldelli Bombelli F., Dawson K.A. (2011). Physical−Chemical Aspects of Protein Corona: Relevance to in Vitro and in Vivo Biological Impacts of Nanoparticles. J. Am. Chem. Soc..

[41] Ellis L.-J.A., Baalousha M., Valsami-Jones E., Lead J.R. (2018). Seasonal variability of natural water chemistry affects the fate and behaviour of silver nanoparticles. Chemosphere.

[42] Ellis L.-J.A., Lynch I. (2020). Mechanistic insights into toxicity pathways induced by nanomaterials in Daphnia magna from analysis of the composition of the acquired protein corona. Environ. Sci. Nano.

[43] Ostermeyer A.-K., Kostigen Mumuper C., Semprini L., Radniecki T. (2013). Influence of Bovine Serum Albumin and Alginate on Silver Nanoparticle Dissolution and Toxicity to Nitrosomonas europaea. Environ. Sci. Technol..

[44] Levak M., Burić P., Dutour Sikirić M., Domazet Jurašin D., Mikac N., Bačić N., Drexel R., Meier F., Jakšić Ž., Lyons D.M. (2017). Effect of Protein Corona on Silver Nanoparticle Stabilization and Ion Release Kinetics in Artificial Seawater. Environ. Sci. Technol..

[45] Peretyazhko T.S., Zhang Q., Colvin V.L. (2014). Size-Controlled Dissolution of Silver Nanoparticles at Neutral and Acidic pH Conditions: Kinetics and Size Changes. Environ. Sci. Technol..

[46] Lee W.S., Kim E., Cho H.-J., Kang T., Kim B., Kim M.Y., Kim Y.S., Song N.W., Lee J.-S., Jeong J. (2018). The Relationship between Dissolution Behavior and the Toxicity of Silver Nanoparticles on Zebrafish Embryos in Different Ionic Environments. Nanomaterials.

[47] Groh K.J., Dalkvist T., Piccapietra F., Behra R., Suter M.J.-F., Schirmer K. (2015). Critical influence of chloride ions on silver ion-mediated acute toxicity of silver nanoparticles to zebrafish embryos. Nanotoxicology.

[48] Qiang L., Arabeyyat Z.H., Xin Q., Paunov V.N., Dale I.J.F., Lloyd Mills R.I., Rotchell J.M., Cheng J. (2020). Silver Nanoparticles in Zebrafish (Danio rerio) Embryos: Uptake, Growth and Molecular Responses. Int. J. Mol. Sci..

[49] Hubbell J.A., Thomas S.N., Swartz M.A. (2009). Materials engineering for immunomodulation. Nature.

[50] Kettler K., Veltman K., van de Meent D., van Wezel A., Hendriks A.J. (2014). Cellular uptake of nanoparticles as determined by particle properties, experimental conditions, and cell type. Environ. Toxicol. Chem..

[51] Buono C., Anzinger J.J., Amar M., Kruth H.S. (2009). Fluorescent pegylated nanoparticles demonstrate fluid-phase pinocytosis by macrophages in mouse atherosclerotic lesions. J. Clin. Investig..

[52] Gu J., Xu H., Han Y., Dai W., Hao W., Wang C., Gu N., Xu H., Cao J. (2011). The internalization pathway, metabolic fate and biological effect of superparamagnetic iron oxide nanoparticles in the macrophage-like RAW264.7 cell. Sci. China Life Sci..

[53] Pricop D. (2015). Maria Andrieş Endocytosis and Exocytosis of Gold Nanoparticles. Rom. J. Biophys..

[54] Kuhn D.A., Vanhecke D., Michen B., Blank F., Gehr P., Petri-Fink A., Rothen-Rutishauser B. (2014). Different endocytotic uptake mechanisms for nanoparticles in epithelial cells and macrophages. Beilstein J. Nanotechnol..

[55] Cifuentes-Rius A., Desai A., Yuen D., Johnston A.P.R., Voelcker N.H. (2021). Inducing immune tolerance with dendritic cell-targeting nanomedicines. Nat. Nanotechnol..

[56] Tomić S., Đokić J., Vasilijić S., Ogrinc N., Rudolf R., Pelicon P., Vučević D., Milosavljević P., Janković S., Anžel I. (2014). Size-Dependent Effects of Gold Nanoparticles Uptake on Maturation and Antitumor Functions of Human Dendritic Cells In Vitro. PLoS ONE.

[57] Zolnik B.S., González-Fernández A., Sadrieh N., Dobrovolskaia M.A. (2010). Nanoparticles and the immune system. Endocrinology.

[58] Luo Y.-H., Chang L.W., Lin P. (2015). Metal-Based Nanoparticles and the Immune System: Activation, Inflammation, and Potential Applications. BioMed Res. Int..

[59] Turvey S.E., Broide D.H. (2010). Innate immunity. J. Allergy Clin. Immunol..

[60] ten Broeke T., Wubbolts R., Stoorvogel W. (2013). MHC class II antigen presentation by dendritic cells regulated through endosomal sorting. Cold Spring Harb. Perspect. Biol..

[61] Hochweller K., Wabnitz G.H., Samstag Y., Suffner J., Hämmerling G.J., Garbi N. (2010). Dendritic cells control T cell tonic signaling required for responsiveness to foreign antigen. Proc. Natl. Acad. Sci. USA.

[62] Lanier L.L. (2004). Nk Cell Recognition. Annu. Rev. Immunol..

[63] Vivier E., Malissen B. (2005). Innate and adaptive immunity: Specificities and signaling hierarchies revisited. Nat. Immunol..

[64] Wang R., Jaw J.J., Stutzman N.C., Zou Z., Sun P.D. (2012). Natural killer cell-produced IFN-γ and TNF-α induce target cell cytolysis through up-regulation of ICAM-1. J. Leukoc. Biol..

[65] Roda J.M., Parihar R., Magro C., Nuovo G.J., Tridandapani S., Carson W.E. (2006). Natural Killer Cells Produce T Cell–Recruiting Chemokines in Response to Antibody-Coated Tumor Cells. Cancer Res..

[66] Fauriat C., Long E.O., Ljunggren H.-G., Bryceson Y.T. (2010). Regulation of human NK-cell cytokine and chemokine production by target cell recognition. Blood.

[67] Swain S.L., McKinstry K.K., Strutt T.M. (2012). Expanding roles for CD4+ T cells in immunity to viruses. Nat. Rev. Immunol..

[68] Schuemann J., Bagley A.F., Berbeco R., Bromma K., Butterworth K.T., Byrne H.L., Chithrani B.D., Cho S.H., Cook J.R., Favaudon V. (2020). Roadmap for metal nanoparticles in radiation therapy: Current status, translational challenges, and future directions. Phys. Med. Biol..

[69] Penninckx S., Heuskin A.-C., Michiels C., Lucas S. (2020). Gold Nanoparticles as a Potent Radiosensitizer: A Transdisciplinary Approach from Physics to Patient. Cancers.

[70] Penninckx S., Heuskin A.-C., Michiels C., Lucas S. (2018). The role of thioredoxin reductase in gold nanoparticle radiosensitization effects. Nanomedicine.

