# Supplementary material for: Mass Cytometry Exploration of Immunomodulatory Responses of Human Immune Cells Exposed to Silver Nanoparticles
Source: Pharmaceutics. 2022 Mar 12;14(3):630. doi: 10.3390/pharmaceutics14030630 (PMC8954471; doi:10.3390/pharmaceutics14030630)
Supplement: Supplementary file 1 [file pharmaceutics-14-00630-s001.zip › pharmaceutics-1571411-supplementary.pdf]

pharmaceutics

Supplementary Materials: Mass Cytometry Exploration of Size-Dependent Immunomodulatory Responses of Human Immune Cells Exposed to Silver Nanoparticles

Jiwon Bae, My Kieu Ha, Haribalan Perumalsamy, Jaewoo Song and Tae-Hyun Yoon

SUPPLEMENTARY METHODS

Core size characterization

Core sizes of the silver nanoparticles (AgNPs) were characterized by transmission electron microscopy (TEM). AgNPs were dispersed in deionized (DI) water and sonicated for 30 minutes. A droplet of each AgNP dispersion was then aliquoted onto a 300-mesh carbon-coated copper grid (Electron Microscopy Sciences, USA) and allowed to air-dry. Images of approximately 1,000 particles were acquired on transmission microscope JEM-1400 Flash (JEOL Ltd., Japan) at 120 kV. Core size distributions were analyzed using the ImageJ software (NIH, USA).

Hydrodynamic size and zeta potential characterization

Dispersions of AgNPs were prepared in DI water and RPMI complete media, which was RPMI-1640 medium (LonzaTM BioWhittakerTM, USA) supplemented with 10% fetal bovine serum (Gibco, USA) and 1% penicillin/streptomycin (Gibco, USA). The hydrodynamic sizes and zeta potentials were measured using a Malvern Zetasizer (Nano-ZS, Malvern Instruments Ltd, UK) following manufacturer' instructions. Measurements were performed in triplicate.

Dissolution measurement

Dissolution of the AgNPs after being dispersed in DI water and RPMI media for 0 and 3 hours was measured by ICPMS. The AgNPs were diluted in DI water and RPMI media to make 0.5 mL dispersions, which were then transferred to centrifugal filters and centrifuge for 30 minutes at 12,000 × *g*. The initial concentration of Ag NPs was 100 μg/mL. The filtered solutions were further diluted in 3% nitric acid and used for ICPMS (NexION 300D, PerkinElmer Inc., USA) measurements to quantify the dissolved Ag^+ content. Three replicates were prepared for each Ag NP sample. The Ag^+ ion standard samples (N9300233, PerkinElmer Inc., USA) were used to generate a calibration curve for quantitative measurements. Based on the calibration curve and the measured ICPMS intensities, the dissolved Ag^+ content was determined, while the dissolution ratio was calculated as

$[Ag^+]/[Ag\ NPs]^{initial} \times 100\%$.

.

SUPPLEMENTARY FIGURES

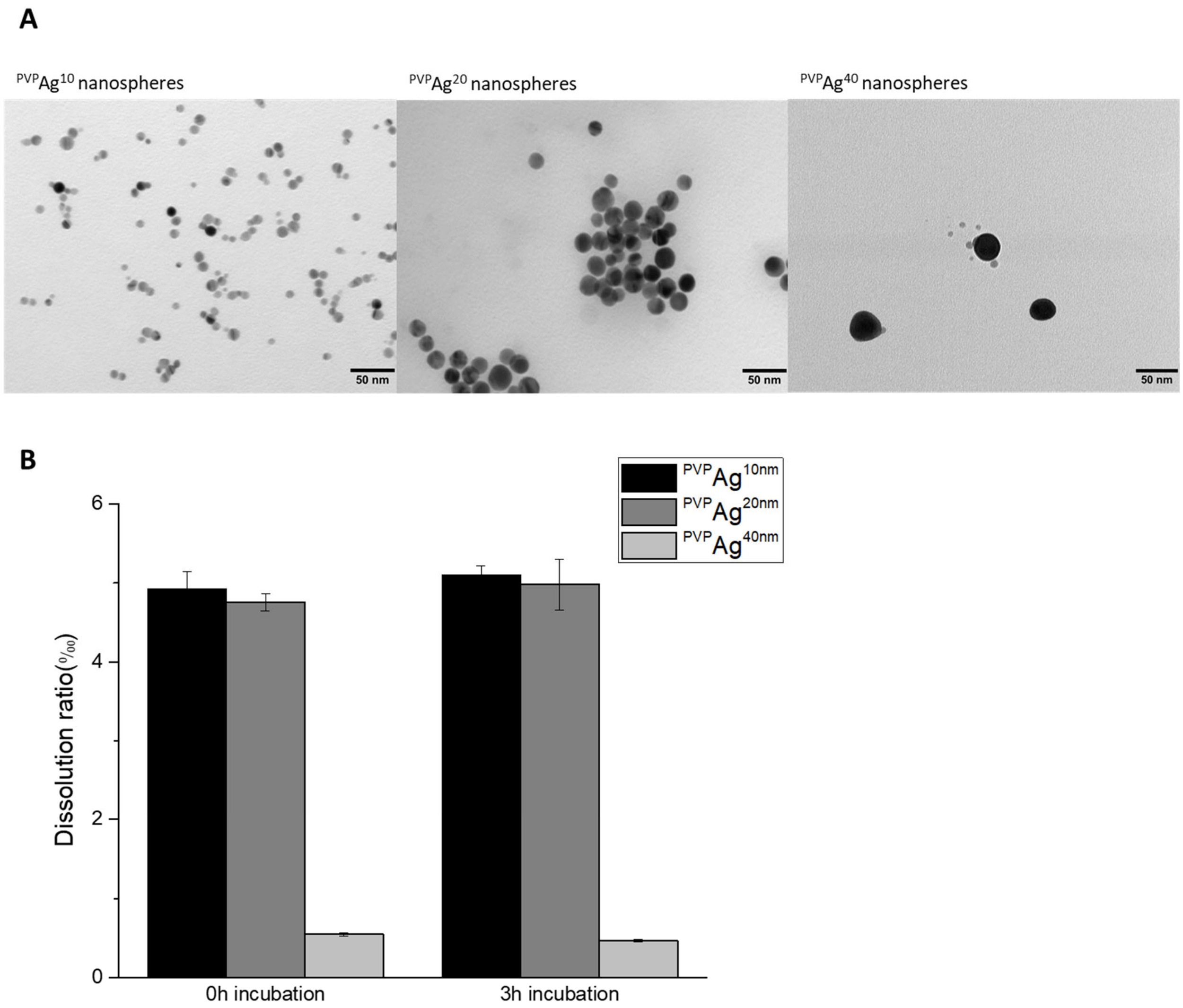

Figure S1. Physicochemical characteristics of $^{PVP}Ag^{10}$, $^{PVP}Ag^{20}$, and $^{PVP}Ag^{40}$. (**A**) TEM images of the Ag NPs. The size of scale bars is 50 nm. (**B**) Dissolution ratios of Ag NPs in RPMI media with 10% FBS.

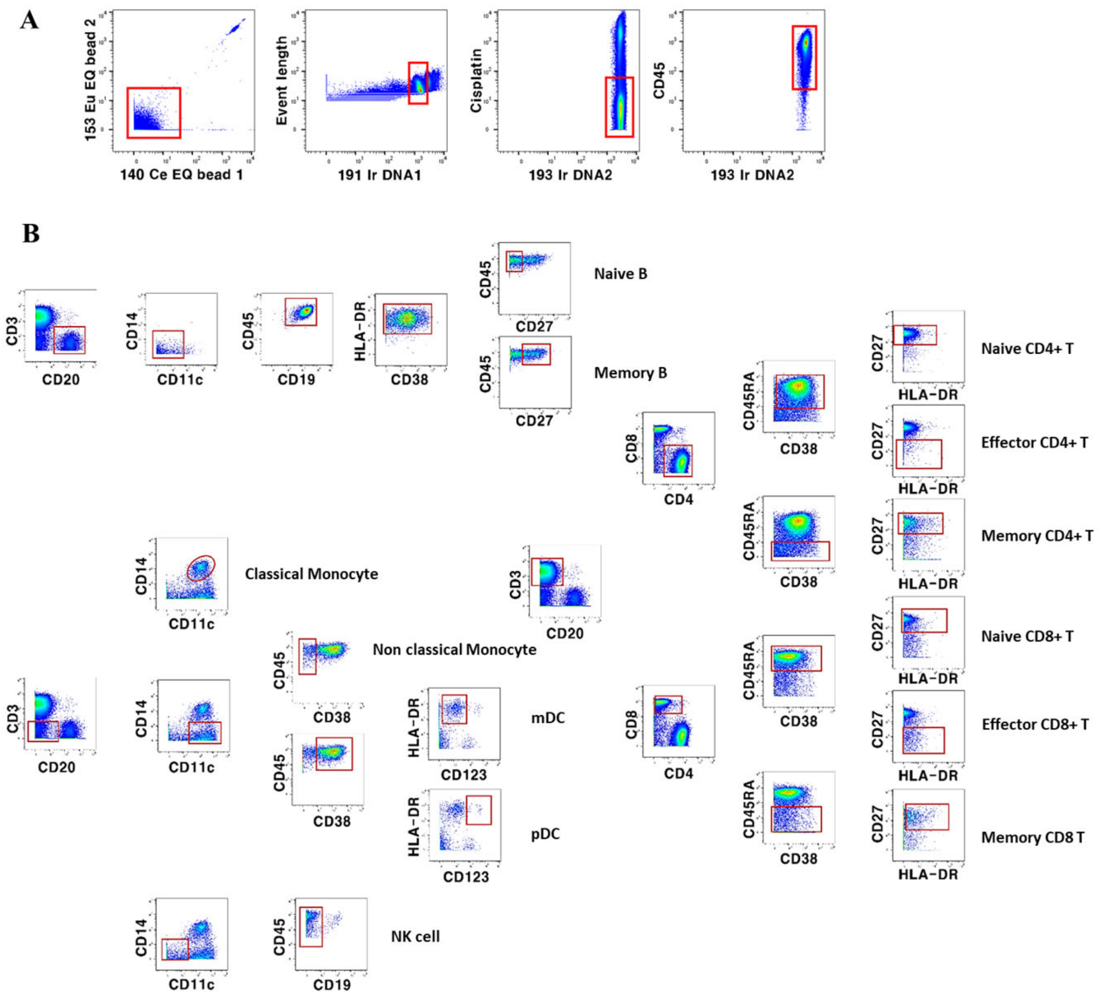

Figure S2. Manual gating strategies for identifying the major immune cell types. Manual gating strategies for (**A**) preprocessing and (**B**) major immune cell phenotyping. The gating strategy pipeline and surface markers are used to identify the specific immune cell types respectively.

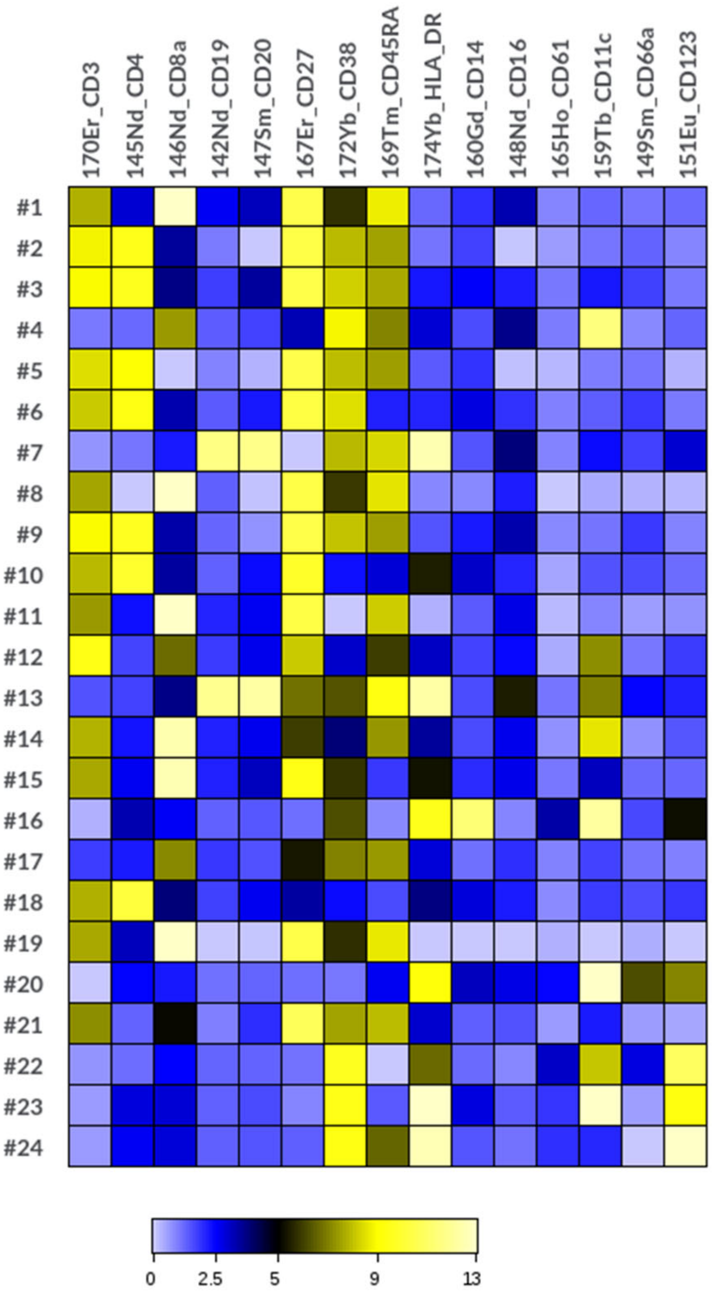

Figure S3. Intensity of phenotypic surface markers expression. The heat map represents surface markers expression of phonograph clusters of manually gated population.

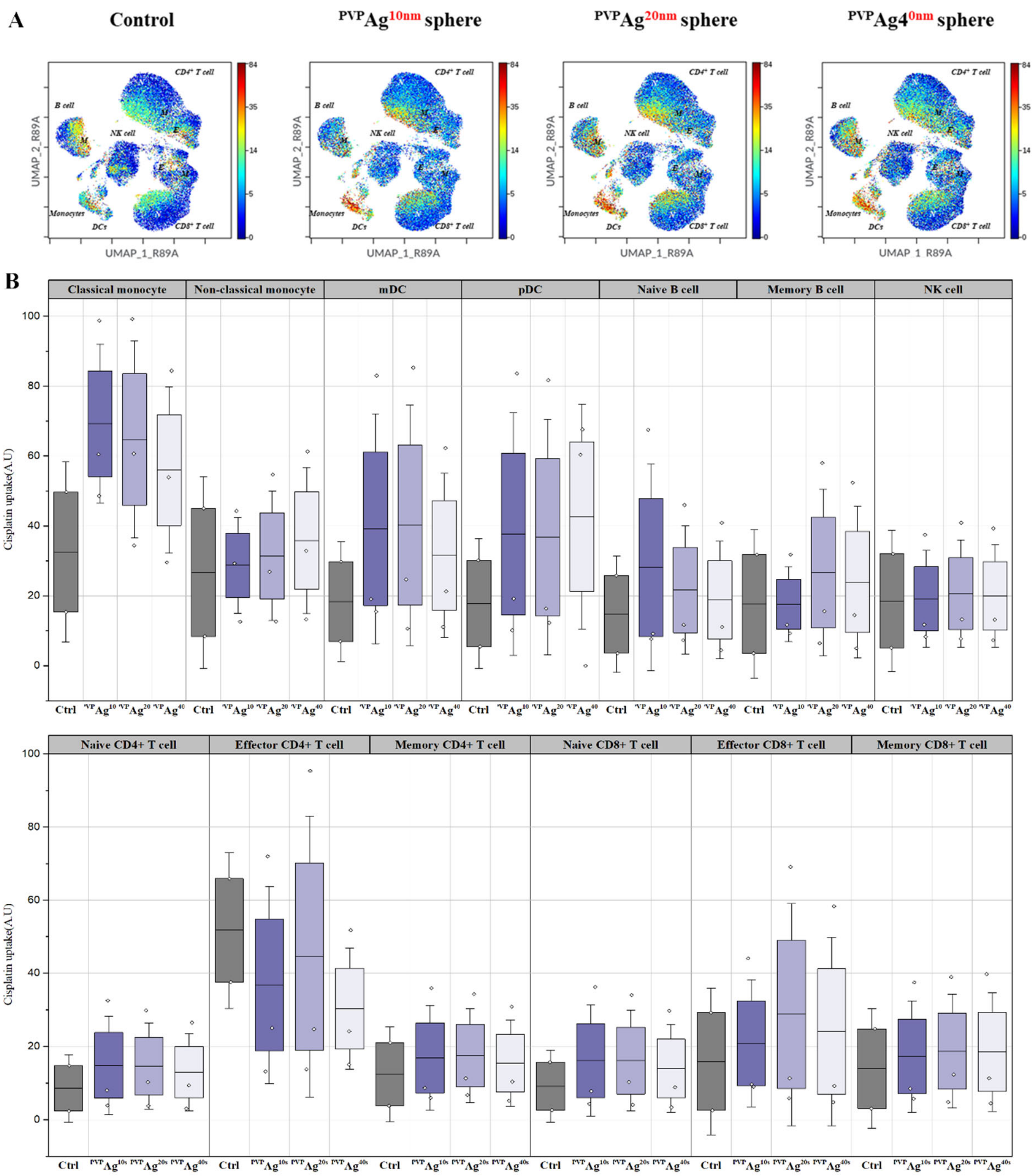

Figure S4. (**A**) ^{195}Pt intensity distributions in UMAP plots for the control and exposed samples treated with PVPAg10, PVPAg20, and PVPAg40. (**B**) The cell viability was observed in different immune cell types by cisplatin uptake following exposure of Ag NPs.

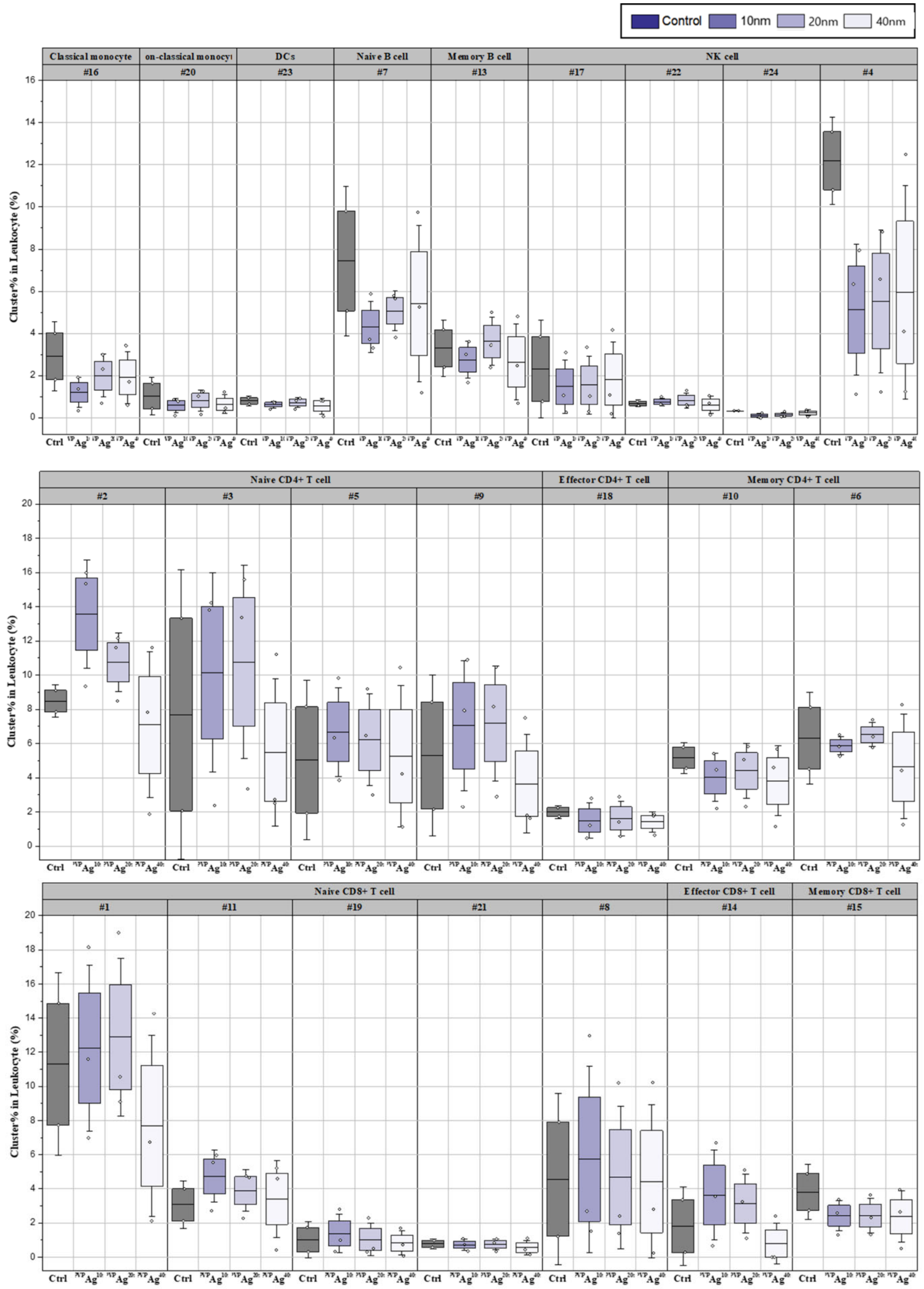

Figure S5. Immune cells subsets population of Population abundance of Phenograph clusters.

SUPPLEMENTARY TABLES

Table S1. Hydrodynamic diameters and zeta potentials of the Ag NPs in deionized water and RPMI media with 10% FBS.

**Nanoparticles**	**Diameter [nm]**		**Zeta potential [mV]**	
	**In DI Water (Core Size)**	**In RPMI Media (Hydrodynamic Size)**	**In DI Water**	**In RPMI Media**
$^{PVP}Ag^{10}$ nanospheres	10 ± 2	41 ± 13	-31.4 ± 1.1	-8.3 ± 0.1
$^{PVP}Ag^{20}$ nanospheres	21 ± 4	75 ± 9	-27.4 ± 1.6	-8.6 ± 1.7
$^{PVP}Ag^{40}$ nanospheres	39 ± 4	77 ± 33	-17.2 ± 1.1	-7.7 ± 0.9

Table S2. List of metal-tagged antibodies of surface markers and intracellular cytokine markers.

**Surface markers**					
Target	Clone	Metal	Target	Clone	Metal
CD3	UCHT1	170Er	CD38	HIT2	172Tb
CD4	RPA-T4	145Nd	CD45	HI30	154Sm
CD8a	RPA-T8	146Nd	CD45RA	HI200	169Tm
CD11c	Bu15	159Tb	CD61	VI-PL2	165Ho
CD14	M5E2	160Gd	CD66	CD66a-B1.1	149Sm
CD16	3G8	148Nd	CD123	6H6	151Eu
CD19	HIB19	142Nd	CD27	O323	167Er
CD20	2H7	147Sm	HLA-DR	L243	174Yb
**Cytokine marker**					
Target	Clone	Metal	Target	Clone	Metal
IFNγ	B27	168Er	IL-17A	N49-653	164Dy
IL-2	MQ-117H12	158Gd	IL-17F	SHLR17	166Er
IL-4	MP4-25D2	144Nd	Granzyme B	GB11	171Yb
IL-5	TRFK5	143Nd	Perforin	B-D48	175Lu
IL-6	MQ2-13A5	156Gd	MIP1β	D21-1351	150Nd
TNFα	Mab11	152Sm			
